# Threshold values of ankle dorsiflexion and gross motor function in 60 children with cerebral palsy: A cross-sectional study

**DOI:** 10.1080/17453674.2018.1456749

**Published:** 2018-03-28

**Authors:** Helle M Rasmussen, Joachim Svensson, Maria Thorning, Niels W Pedersen, Søren Overgaard, Anders Holsgaard-Larsen

**Affiliations:** a Department of Orthopedic Surgery and Traumatology, Odense University Hospital;; b Department of Clinical Research, University of Southern Denmark, Odense, Denmark

## Abstract

**Background and purpose — Threshold values defining 3 categories of passive range of motion are used in the Cerebral Palsy follow-Up Program to guide clinical decisions. The aim of this study was to investigate the threshold values by testing the hypothesis that passive range of motion in ankle dorsiflexion is associated with gross motor function and that function differs between the groups of participants in each category.**

**Patients and methods — We analyzed data from 60 ambulatory children (aged 5–9 years) with spastic cerebral palsy. Outcomes were passive range of motion in ankle dorsiflexion with flexed and extended knee and gross motor function (Gait Deviation Index, Gait Variable Score of the ankle, peak dorsiflexion during gait, 1-minute walk, Gross Motor Function Measure, the Pediatric Quality of Life Inventory Cerebral Palsy Module, and Pediatric Outcomes Data Collection Instrument).**

**Results — Significant (p < 0.05) and moderate correlations were documented for range of motion versus Gait Variable Score of the ankle (r = –0.37 and r = –0.37) and range of motion versus peak dorsiflexion (r = 0.49 and r = 0.55). Differences between the groups formed by the categories were shown for Gait Variable Score of the ankle and peak dorsiflexion (p < 0.05). No other significant correlations or differences between the categories were observed.**

**Interpretation — The results suggest that threshold values for ankle dorsiflexion used in the Cerebral Palsy follow-Up Program are of limited clinical value in assessing overall gross motor function, but may be used to identify deviations in ankle-specific gait function.**

Muscle contractures and joint deformities are common manifestations of cerebral palsy (CP) (Nordmark et al. 2009). A surveillance program, entitled the Cerebral Palsy follow-Up Program (CPUP), is used to ensure early identification and treatment of deterioration (Rasmussen et al. 2016, Alriksson-Schmidt et al. 2017). As part of the evaluation, the CPUP uses threshold values inspired by traffic light signals on passive range of motion (ROM). The ROM is classified on the basis of threshold values into 3 categories with the following interpretation: “green” means “clear” and that no indication of deterioration is noted, “yellow” indicates that vigilant observation, modified treatment, or initiation of treatment is necessary, and “red” indicates “alert” and that treatment is urgently needed, assuming no contraindications are present (Alriksson-Schmidt et al. 2017). For children on the Gross Motor Function Classification System (GMFCS) levels I–III, the threshold values have been set to ensure that the patient has enough ROM to perform ankle dorsiflexion (DF) in the stance and swing phases of walking (CPOP 2017). The categories are used as easy-to-understand interpretations of the measurement of ROM and are used to guide decisions about future examinations and interventions (Hagglund and Wagner 2011).

A study investigating the association between the 3 categories of ROM in DF and motor function measured with the Functional Mobility Scale found a significant association between the categories (chi-square association, r_Φ_ = –0.27, p = 0.01) in young adults with CP (Brantmark et al. 2015). Despite the use of the threshold values for clinical evaluation in several countries, how these thresholds were identified has never been fully explained and, to our knowledge, they are not evidence-based. Furthermore, their potential relationships with measures of gross motor function in children with CP have never been established.

Thus, the aim of the study was to investigate the threshold values used by CPUP by testing the hypothesis that ROM in DF is associated with gross motor function and that gross motor function differs between the groups of participants in each category. Gross motor function is measured by various methods describing the overall gross motor capacity, the ankle-specific gait capacity, and the use of gross motor skills in everyday life.

## Patients and methods

We performed a cross-sectional study based on data from the baseline assessment in a randomized controlled trial (Rasmussen et al. 2015b)/NCT02160457. The reporting of the current study conforms to recommendations by the STROBE panel.

GMFM: Gross Motor Function Measure; GVS: Gait Variable Score; IQR: inter-quartile range; Pedsql: the Pediatric Quality of Life Inventory Cerebral Palsy Module; PODCI: Pediatric Outcomes Data Collection Instrument; ROM: range of motion; SD: standard deviation.

### Participants and setting

Patients registered in the Danish version of the CPUP in the Region of Southern Denmark and the North Denmark Region were screened for eligibility and invited to participate. Eligibility criteria have been described in detail previously (Rasmussen et al. 2015b). In brief, eligible participants were children aged 5 to 8 years, diagnosed with spastic CP at GMFCS levels I or II. Children were not eligible if they had received earlier interventions in the form of orthopedic surgery in the previous 52 weeks or injection with botulinum toxin in the 12 weeks prior to the baseline assessment (exclusion criteria). Furthermore, children were excluded if they were unable to demonstrate sufficient cooperation and cognitive understanding to participate in the examination, if they relocated to another region during the trial, or if their parents could not speak and understand Danish.

Participants were recruited and data collected from June 2014 until July 2016. Questionnaires were mailed to the parents prior to the examination at the Motion Analysis Laboratory at Odense University Hospital. 6 experienced physiotherapists, who performed each examination in pairs, were involved in data collection.

### Measurements

The baseline assessment methodology has previously been described in detail (Rasmussen et al. 2015b). In short, the assessment consisted of: patient characteristics, a thorough physical examination, 3-D instrumented gait analysis, functional tests of walking and gross motor capacity, and patient-reported outcomes concerning the use of gross motor skills in everyday life (see below).

Patient characteristics were described using the following measures: sex, age (years), height (meters), weight (kilograms), CP subtype (unilateral or bilateral spastic CP), and classification according to the GMFCS.

Classification according to the 3 categories was obtained from measurements of maximal DF with flexed knee (DF (knee 90°)) and extended knee (DF (knee 0°)) performed by 2 examiners with a goniometer according to the CPUP protocol (2017). The starting positions were supine, hip and knee in 90° of flexion when measuring DF (knee 90°), or with the hip and knee extended when measuring DF (knee 0°). While the hind foot was maintained in neutral to avoid calcaneal valgus or varus, the fixed arm of the goniometer was placed parallel to the front of the tibia and the moving arm at the lateral side of the foot (Nordmark et al. 2009). The threshold values for DF used by the CPUP are outlined in Table 1.

**Table 1.** Patient characteristics

Girls/boys	21/39
Age (years, months), mean (SD)	6y 10m (1y 3m)
Height (meters), mean (SD)	1.23 (0.1)
Weight (kg), mean (SD)	24 (6.8)
Cerebral palsy subtype and function:	
CP spastic subtype, unilateral/bilateral	43/17
GMFCS levels I/II	42/18
Passive range of motion:	
Dorsiflexion with knee 90°, mean (SD)	21 (11)
Dorsiflexion with knee 0°, mean (SD)	14 (10)
Ankle ROM categories	
Dorsiflexion (knee 90°), participants, n	
Red: range ≤10°	14
Yellow: range 10°–20°	6
Green: range ≥20°	40
Dorsiflexion (knee 0°), participants, n	
Red: range ≤0°	14
Yellow: range 0°–10°	5
Green: range ≥10°	49
Gait summary measures and peak dorsiflexion:	
GDI score, mean (SD)	76 (13)
GVS ankle, median (IRQ)	7.9 (6.6)
Peak dorsiflexion (degrees), mean (SD)	13 (6.5)
Gross motor capacity and performance:	
1-min walk test (meters), mean (SD)**^a^**	64 (11)
GMFM, mean (SD)	82 (8.4)
Pedsql movement and balance, mean (SD)	75 (19)
PODCI transfer and basic mobility, median (IRQ)	93 (15)

**^a^** Data are available for only 57 participants.

Abbreviations: CP: cerebral palsy; GDI: Gait Deviation Index; GMFCS: Gross Motor Function Classification System;

The method used for data collection with 3-D instrumented gait analysis has previously been described (Rasmussen et al. 2015b). Briefly, we collected 10 walking trials at a self-selected speed using an 8-camera motion capture system (Vicon MX03, Oxford, UK) and the Plug-in-Gait model (Davis et al. 1991). Vicon Nexus software (version 1.7.1-1.8.5) and Vicon Polygon software (version 3.5.2-4.3) were used for data processing to deﬁne gait cycles for 10 trials from each participant. Subsequently, 5 trials with a consistent velocity (±15%) were selected and used for the calculation of the Gait Deviation Index (GDI), Gait Variable Score (GVS) of the ankle, and the maximal active DF in the stance phase (Schwartz and Rozumalski 2008, Baker et al. 2009). The GDI and GVS of the ankle are gait summary measures of overall gait function and ankle joint kinematics, providing information on the deviation from a reference group. For reference, we used our own dataset of 30 typically developing children (Rasmussen et al. 2015a). A reliability study performed in our laboratory has documented excellent intra-rater reliability and acceptable agreement for the GDI and fair to good intra-rater reliability and acceptable agreement for the GVS across 2 repeated sessions in children with CP (Rasmussen et al. 2015a).

Gross motor capacity was assessed using the 1-minute walk test (McDowell et al. 2009) and selected items from the 66-item Gross Motor Function Measure (Russell et al. 2013). The use of gross motor skills in everyday life was assessed using a Danish version of the subscale movement and balance of the Pediatric Quality of Life Inventory Cerebral Palsy Module (Varni et al. 2006) and a Danish version of the subscale of transfer and basic mobility of the Pediatric Outcomes Data Collection Instrument (Daltroy et al. 1998).

### Statistics

The current study is based on a sample of children with CP who volunteered to participate in a randomized controlled trial. The sample size calculation for the original study was based on a between-group change score of 7.9 points on the primary outcome measure: the GDI (Rasmussen et al. 2015b).

Descriptive statistics were calculated for sex, CP subtype, and classification according to the GMFCS. In the statistical analysis, the median scores of the GDI and GVS of the ankle from 5 trials were used. In the analysis of ROM, the GDI, GVS of the ankle, and Peak dorsiflexion we used data from the affected side of patients with unilateral CP, and for participants with bilateral CP we used the most affected side. The most affected side was determined as the leg with the highest number of measurements in the red and/or yellow categories in DF and in cases without differences in categories, the side with the lowest GDI. The statistical distribution of data was investigated using normal probability plots and the Shapiro–Wilk test. The GVS of the ankle (p < 0.001) and Pediatric Outcomes Data Collection Instrument transfer and basic mobility scores (p < 0.001) were not normally distributed.

Scatterplots with fitted values of the outcome measures were prepared to provide an overview of the data. Correlations between ROM and the outcome variables were investigated with Pearson correlation coefficients, except for the GVS of the ankle and Pediatric Outcomes Data Collection Instrument transfer and basic mobility scores, where Spearman’s rank correlation coefficients were used. The correlation coefficients were interpreted according to Dancey and Reidy (2011). Differences in the normally distributed outcome variables in the 3 ROM categories were investigated with one-way ANOVA. The GVS of the ankle and Pediatric Outcomes Data Collection Instrument transfer and basic mobility scores were both assessed with the Kruskal–Wallis test and, if relevant, pairwise comparisons with the Wilcoxon rank-sum test (Mann-Whitney).

Statistical analyses were performed using Stata/IC 14.2 for Mac (StataCorp, College Station, TX, USA). The significance level for all statistical results was p < 0.05.

### Ethics, registration, funding, and potential conflicts of interest

The study complies with the principles of the Declaration of Helsinki. Study approval was obtained from the Committee for Medical Research Ethics in the Region of Southern Denmark (S-20120162) and the Danish Data Protection Agency (2008-58-0035). Informed consent to participate was achieved. Trial registration: ClinicalTrials.gov NCT02160457.

The study was funded by the University of Southern Denmark; Odense University Hospital Research grants, the Region of Southern Denmark Research and PhD grants, the Physiotherapy Praxis Foundation, Ludvig and Sara Elsass Foundation, and the Danish Physiotherapy Research Fund. The funding organizations had no involvement in the study design; in collection, analysis, and interpretation of the data; in the writing of the report; or in the decision to submit the article for publication.

All the authors of this manuscript declare that they have no conflict of interest related to the current study.

## Results

160 patients were invited to participate in the randomized controlled trial. Of these, 48 patients did not answer, 36 were not interested in further information and 16 were not eligible after screening. Consequently, this cross-sectional study was based on 60 participants with spastic CP at GMFCS levels I and II (21 girls; average age 6 years and 10 months (SD 1 year 3 months), 43 diagnosed with unilateral CP) (Table 1).

Statistically significant moderate correlations were observed between the GVS of the ankle and DF with flexed knee (r = –0.37, [95% CI –0.57 to –0.13], p < 0.05) and extended knee (r = –0.37, [CI –0.57 to –0.13], p < 0.05) and peak dorsiflexion and DF with flexed knee (r = 0.49, [CI 0.26–0.67], p < 0.001) and extended knee (r = 0.55, [CI 0.35–0.71], p < 0.001) (Table 2).

**Table 2.** Correlation coefficients, coefficient of determination, mean/median scores of the outcome measures in the groups formed by their ankle ROM, and results of the ANOVA/Kruskal–Wallis test. Values for ankle ROM are mean (SD) unless otherwise stated

	r	Correlation 95% CI	p-value	Red		Ankle ROM Yellow	Green	Diff. p-value
Dorsiflexion (knee 90°), n				14		6	40	
GDI score	0.12	[–0.14 to 0.36]	0.4	75 (10)		78 (21)	77 (12)	0.9
Peak dorsiflexion	0.49	[0.26 to 0.67]	< 0.001	8.2 (5.8)		15 (7.0)	14 (6.0)	0.007
1-min walk test**^a^**	0.11	[–0.15 to 0.35]	0.4	76 (18)		78 (15)	80 (13)	0.7
GMFM	0.09	[–0.17 to 0.34]	0.5	83 (8.3)		82 (11)	82 (8.2)	1.0
Pedsql	–0.01	[–0.26 to 0.24]	0.96	78 (22)		73 (19)	74 (19)	0.7
GVS ankle ^b^	–0.37	[–0.57 to –0.13]	< 0.05	13 (11)		7.2 (3.7)	7.6 (4.5)	0.03
PODCI ^b^	0.01	[–0.24 to 0.26]	> 0.05	95 (17)		92 (31)	92 (14)	0.7
Dorsiflexion (knee 0°), n		6	5	49				
GDI score	0.05	[–0.21 to 0.30]	0.7	78 (7.6)		71 (18)	77 (13)	0.6
Peak dorsiflexion	0.55	[0.35 to 0.71]	< 0.001	4.9 (4.3)		6.6 (5.6)	14 (5.7)	< 0.001
1-min walk test**^a^**	0.17	[–0.09 to 0.41]	0.2	73 (29)		80 (14)	80 (12)	0.5
GMFM	0.09	[–0.17 to 0.34]	0.5	83 (8.3)		82 (11)	82 (8.2)	1.0
Pedsql	0.06	[–0.20 to 0.31]	0.7	70 (16)		84 (20)	74 (20)	0.5
GVS ankle ^b^	–0.37	[–0.57 to –0.13]	< 0.05	17 (5.2)		16 (13)	7.6 (4.2)	0.02
PODCI ^b^	0.04	[–0.22 to 0.29]	> 0.05	85 (30)		93 (17)	94 (14)	0.8

**^a^** Data available for 57 participants (data are missing for 3 participants in the green category).

**^b^** Ankle ROM values are median (IQR)

Abbreviations: See Table 1.

There were statistically significant differences in the GVS of the ankle and peak dorsiflexion between the 3 groups of participants based on the categories with flexed and extended knee (Table 2). For DF with flexed knee, the median GVSs of the ankle for the red and green categories were 14° and 7.6°; the distributions in the 2 groups differed significantly ((z-score, p-value), z = –2.63, p = 0.009) and with extended knee, the median GVS of the ankle for the red and green categories were 17° and 7.6°; the distributions in the 2 groups differed significantly (z = –2.43, p = 0.02). For peak dorsiflexion, we observed a difference in red versus green and red versus yellow ROM categories with flexed knee ((mean (95% CI) –9.6° (–14 to –4.7) and –7.9° (–13 to –2.6), respectively) and between red versus green and yellow versus green ROM categories with extended knee (–9.6° (–15.4 to –3.8) and –7.9° (–14 to –1.5), respectively). No statistically significant group-mean differences were observed between the participants classified into each of the ROM categories of DF on the variables of GDI, 1-minute walk, Gross Motor Function Measure, the Pediatric Quality of Life Inventory Cerebral Palsy Module, and Pediatric Outcomes Data Collection Instrument transfer and basic mobility scores.

## Discussion

This study aimed to investigate the threshold values of ROM in DF used by the CPUP. We hypothesized that DF and gross motor function would be associated and that there would be differences in gross motor function between the 3 groups based on the categories. We found moderate correlations between DF and deviations in ankle movement during gait in children with CP at GMFCS levels I and II. Furthermore, we found differences between scores of the specific gait function in the ankle joint (GVS of the ankle and peak dorsiflexion) and the 3 ROM categories but no association or differences were observed for overall measures of gross motor capacity (GDI, 1-minute walk, and Gross Motor Function Measure) or the use of gross motor skills in everyday life. Thus, our hypotheses were only partly confirmed and the results suggest that threshold values of DF in the CPUP are of limited clinical value in assessing overall gross motor capacity and the use of gross motor skills in everyday life, but may be used to identify deviations in ankle-specific gait function. Detection of deviations in ankle-specific gait function might be useful in the identification of distal deterioration before it might progress to more proximal involvement.

Our findings accord with the relationship between changes in passive ROM and gait function from a research project investigating the effects of gastrocnemius fascia lengthening in 19 children with CP (mean age 8 years) on DF by goniometry and gait function by gait summary measures (Galli et al. 2005). That study reported improvements in DF with flexed knee (from 4.3° before to 8.6°after surgery) and extended knee (from –4.3° before to 9.4° after surgery) with accompanying improvements in overall gait function (GDI from 70 before to 83 after surgery) (GVS of the ankle from 22° before to 12°after surgery). These findings suggest that improvements in DF entail improvement in gait function at the joint level (GVS of the ankle) and, to some degree, in overall gait function (GDI).

The moderate correlations we found suggest that factors other than ROM may explain the majority of the observed variation. This is supported by a study comparing clinical examination (passive ROM, spasticity, strength, and selective motor control) with 3-D instrumented gait analysis in children with CP (Desloovere et al. 2006). That study found a fair to moderate correlation between the clinical examination and data from the 3-D instrumented gait analysis and concluded that both types of data provide important information on the problems faced by children with CP.

The categories used in the CPUP are set to ensure that the patient has enough ROM to perform adequate DF in walking (CPOP 2017). Our results support the complex interaction between different dimensions of function, as proposed by the International Classification of Functioning, Disability and Health (WHO 2007). However, it might be important to investigate the categories for other important issues of spastic gait, such as energy expenditure or the risk of developing deformities (Hagglund et al. 2005, Nordmark et al. 2009).

To ensure valid data from the 3-D instrumented gait analysis we decided not to include children at GMFCS level III. However, to promote a representative sample of young children with spastic CP at GMFCS levels I and II, our inclusion and exclusion criteria were kept open. Thus, the study sample is not representative of the total population of children with CP and only a few participants experienced ankle ROM affected above red threshold values, which can be caused by the fact that reduced ROM usually first arises later in life ­(Nordmark et al. 2009). Thus, there remains a need to investigate the ROM categories in older children, with possibly more reduced ROM. Furthermore, children on higher levels of the GMFCS and ROM categories for the other joints of the lower extremities should also be investigated. Finally, it would be important to investigate whether certain subgroups (i.e. CP subtype, weight, or age) behave differently according to ROM thresholds but due to a relatively small study sample this was not possible.

A design limitation of this cross-sectional study is that data collection was performed only during one session and therefore no conclusions regarding causality can be inferred. Furthermore, the strength of our results is limited by the small sample size. In addition, measurement errors of 10–15° of goniometric measurements of ROM have been reported ­(Nordmark et al. 2009). A study using the Generalizability Theory has shown a measurement error in DF of 9° in between-day measurements, when performed by 3 physiotherapists (McDowell et al. 2000). Due to an inclusion period of 25 months and changes in staff, 6 physiotherapists were involved in data collection. All physiotherapists underwent training in the research protocol. However, we did not investigate the consistency of their measurements, therefore this may have increased the variability of the measurements and, thus, must be seen as a limitation of the study.

In summary, our study found that DF is associated with ankle-specific measures of gross motor function (GVS of the ankle and peak dorsiflexion) and that the mean scores of the ankle-specific measures are different in the 3 groups based on the categories. In contrast to our hypothesis, we did not find an important relationship between DF and the 3 related categories to overall measures of gross motor capacity and the use of gross motor skills in everyday life in young children at GMFCS levels I and II.

The implications of our findings suggest that the current threshold values of DF used in the CPUP are of limited clinical value for assessing overall gross motor function, but may be used to identify isolated deviation of ankle function during gait. As a consequence, other measures that are more related to gait function should be considered in the identification of children at risk of functional decline and who may benefit from interventions.

All the authors participated in the concept and design of the study. HMR and AHL were involved in drafting the manuscript. JS, NWP, MT, and SO revised the first draft and commented on and revised the subsequent draft.

The authors would like to thank all participants in the project; physiotherapists Lotte Jensen, Rasmus Sørensen, Line Kiilerich, and Christina Fonvig for helping with data collection and Suzanne Capell for English-language proofreading of the manuscript.

*Acta* thanks Jacques Riad and other anonymous reviewers for help with peer review of this study.
